# Analysis of the Dual-Functional Broadband Properties of an Asymmetric Piezoelectric Metamaterial Beam for Simultaneous Vibration Reduction and Energy Harvesting

**DOI:** 10.3390/ma18215003

**Published:** 2025-11-01

**Authors:** Xingguo Wang, Qiuju Xie, Lan Wang, Haisheng Shu, Hongyan Wang

**Affiliations:** 1 College of Electrical Engineering and Information, Northeast Agricultural University, Harbin 150030, China; wangxingguo2022@163.com; 2 College of Mechanical and Electrical Engineering, Qiqihar University, Qiqihar 161006, China; wanghongyan1993@163.com; 3 College of Mechanical and Electrical Engineering, Harbin Engineering University, Harbin 150001, China; wanglan707@126.com; 4 Electromechanical and Automotive Department, Chizhou Vocational and Technical College, Chizhou 247100, China; shuhaisheng@126.com

**Keywords:** piezoelectric metamaterial, vibration reduction, energy harvesting, broadband, dual function

## Abstract

This paper investigates the dual-functional broadband properties of an asymmetric piezoelectric metamaterial beam for simultaneous vibration reduction and energy harvesting. Firstly, a grading method is proposed, and an asymmetric piezoelectric metamaterial beam structure model with the gradient mode is established. The effects of various gradient modes on the grading parameters of each segment are examined. Subsequently, the band structure and group velocity of each segment are examined to elucidate the propagation and energy harvesting mechanisms for the bending-dominated wave. Furthermore, the evaluation criteria for dual-functional properties in the gradient mode are introduced, revealing the broadening law of the dual-functional band under various gradient modes. Finally, the theoretical results are analyzed and compared with the finite element method (FEM). The results show that in gradient mode, the bending-dominated wave in the asymmetric piezoelectric metamaterial beam generates the spatial frequency division and enhances wave field energy. Compared with the uniform mode, the gradient modes can simultaneously achieve dual-functional effects in both the low-frequency and broadband ranges, significantly improving performance. Parameters such as gradient modes and grading variation ranges significantly impact the dual-functional performance. By reasonably selecting the grading parameters, enhanced dual-functional performance can be achieved.

## 1. Introduction

Metamaterials are a class of designable structured materials that have emerged in the 21st century, exhibiting a series of unique physical properties not found in natural materials, such as negative refractive index [1], negative mass density [2], and negative stiffness [3]. They represent the frontiers of physics, materials science, engineering, and other disciplines. Smart materials, such as piezoelectric materials [4] and magneto- rheological elastomer materials [5], demonstrate characteristics where their properties are regulated by external physical fields. The research on metamaterials originated from the electromagnetic field and gradually extended to vibroacoustic fields and energy harvesting. By leveraging the bandgap properties [6,7], waveguiding capabilities [8,9], and wave-focusing effects [10,11] of metamaterials alongside the piezoelectric effect of smart materials, researchers have conducted relevant studies in vibration/noise reduction and energy harvesting, achieving significant results. More recently, some scholars have focused on the dual-function properties of metamaterials—specifically the simultaneous implementation of vibration/noise suppression and energy harvesting [12,13]. These studies have advanced developments in vibration/noise reduction and energy harvesting.

As early as 2009, Liu et al. [14] pioneered the integration of metamaterials and energy harvesting. They proposed a dual-functional metamaterial system that embedded piezoelectric cantilever beams within a honeycomb structure. Their study demonstrated that the metamaterial with mechanical local oscillators can generate mid-to-low- frequency bandgaps, in which elastic waves are attenuated. The reason is that when the vibrating frequency is near the resonant frequency of the local oscillators, the oscillators exhibit anti-phase motion relative to the substrate, which can lead to the transfer of mechanical energy from the substrate to the oscillator units. Thus, effective localized energy collection becomes feasible by incorporating energy harvesting elements into the local oscillators. Building upon this work, some researchers investigated dual-functional metamaterials with mechanical local oscillators. The studies on both linear [15,16,17,18,19,20,21,22,23,24] and nonlinear designs [25,26,27,28,29,30,31,32] yield significant advancements, which deepen our understanding of multifunctional metamaterial applications.

Unlike metamaterials with mechanical localized oscillators, metamaterials with piezoelectric localized oscillators can achieve a resonant effect through the resonant circuits constituted by inductance elements and the inherent capacitance of piezoelectric bimorphs. As early as 2001, Thorp et al. [4] pioneered the integration of piezoelectric oscillators into rod structures by constructing a one-dimensional periodic array of piezoelectric elements, which is called the piezoelectric metamaterial rod. This study investigated the attenuation and wave localization characteristics of longitudinal waves within the periodic structure and demonstrated a novel approach for bandgap control without altering the host structure. In 2005, Thorp et al. [33] extended this concept to liquid-filled cylindrical shells by periodically arranging piezoelectric rings interconnected with resonant circuits on the inner surface along the axial direction, systematically analyzing the propagation and attenuation behavior of elastic waves in such configurations. In 2009, Ruzzene et al. [34,35] further advanced this framework by developing a two-dimensional piezoelectric periodic array in plates and examining its vibration and wave propagation properties. Building upon these foundational contributions, numerous researchers have carried out extensive investigations on vibration attenuation capabilities of piezoelectric metamaterials, including computation algorithms, formation mechanisms, characteristics of bandgaps, and strategies for bandgap broadening; these efforts yield significant and fruitful outcomes [36,37,38,39,40,41,42,43]. Similarly to the development trajectory of metamaterials with mechanical oscillators, research on single-function piezoelectric metamaterials continues to expand; growing attention has increasingly turned to their dual-functional capabilities.

The representative research efforts in the study of dual-function piezoelectric metamaterials have focused on the Georgia Institute of Technology. In 2017, Erturk et al. [44] conducted the first dual-functional study of vibration reduction and energy harvesting for piezoelectric metamaterials. They developed a bandgap calculation method based on modal analysis. Moreover, these scholars further investigated the energy harvesting performance of the metamaterial beam. The research results indicated that integrating energy harvesting devices into the piezoelectric local oscillators could generate useful electrical energy without affecting the vibration suppression performance of the metamaterial itself. Based on this research, Erturk et al. [45] continued to conduct more in-depth studies on the dual-functional properties of this model in the following year. In 2021, Erturk’s team [46] proposed a piezoelectric metamaterial dual-functional beam based on gradient oscillators, in which the gradient parameter of oscillator units was realized by adjusting inductance parameters in shunt circuits. The results show that all graded modes could broaden the bandwidth of dual-functional action compared with uniform mode piezoelectric metamaterials. Based on these studies, in 2022 Erturk et al. [47] combined mechanical and piezoelectric oscillators to construct a hybrid oscillator metamaterial beam structure. The results indicate that this system’s dual-functional performances have been substantially enhanced compared with uniform mode structures. In addition to Erturk’s team, Tol’s team at the University of Michigan has also targeted dual-function applications in piezoelectric metamaterials. In 2021, Tol’s team [48] introduced a structural model for realizing energy harvesting and attenuation of traveling waves using a piezoelectric metamaterial beam. The results showed that conversion efficiency could reach 95% at local resonance frequency. Based on the above studies, our group has investigated the dual-functional realization mechanism and behavioral properties of the piezoelectric metamaterial beams in stacked and asymmetric modes, which not only enriches the theoretical research framework of the dual-functional structures, but also provide the necessary theoretical basis and technical means for dual-functional applications of the low- and medium-frequency vibration suppression and energy self-supply of microelectromechanical systems [49,50].

Although previous studies have achieved certain research achievements and innovations, some aspects of the research remain to be further explored and deepened. For example, the inherent property of the localized resonant bandgap leads to the relatively narrow dual-functional operating bandwidth for the piezoelectric metamaterial beam. It is essential to conduct further investigations aimed at expanding the operational bandwidth of the dual-functional metamaterial beam. Previous studies have investigated grading design strategies that effectively broaden the bandwidth of piezoelectric metamaterials. The current approach using grading to broaden the bandwidth mainly consists of two forms: namely, grading tuning based on geometric parameters and electrical parameters of piezoelectric elements. Tang et al. [51] introduced a novel gradient piezoelectric metamaterial beam with parallel resonant circuits, employing a graded strategy for electrode pairs’ spatial variation. With proper selection of resistors, the gradient piezoelectric metamaterial beam achieved theoretically the widest attenuation region. On this basis, by setting length-graded piezoelectrics on a beam to construct a piezoelectric metamaterial beam with a spatial grading pattern, Tang et al. [52] discovered that the attenuation zone could be effectively broadened compared with the conventional metamaterial with identical piezoelectric patches. Both of the aforementioned gradient structures belong to the category of geometrically graded piezoelectric elements. Erturk et al. [46] designed a graded metamaterial by attaching piezoelectric arrays connected to different impedances obtained in accordance with a grading strategy to a host structure. Uniformly distributed discrete bandgaps were generated, forming a wide vibration attenuation region. However, this grading approach may result in relatively large inductance parameters for the low-frequency grading units. With the same concept, Liu et al. [53] replaced the resistive-inductive circuit in ref. [46] with an negative capacitance circuit. It is found that the negative capacitance circuit enhanced the electromechanical coupling. A wide attenuation zone was observed in the high-frequency region spanning over 7–11 kHz. Although the negative capacitance technology can effectively enable piezoelectric metamaterials to exhibit broadband characteristics, its active control requirement limits practical applications in this dual-functional context. Therefore, the geometric grading of piezoelectric elements presents certain advantages over graded tuning of circuit parameters.

Indeed, in our prior research, we have demonstrated that introducing asymmetric parameters offers greater flexibility in effectively modulating dual-functional characteristics compared to the conventional symmetrical mode [50]. This enhancement enables the operational frequency band of the dual-functional system to extend into the lower frequency range, thereby achieving superior performance. Inspired by the concept of grading design, this study builds upon the asymmetrical mode piezoelectric metamaterial beam and introduces a gradient structure to the asymmetry of the local oscillator. Without altering the electrical parameters, a gradient mode piezoelectric metamaterial beam is constructed, and its dual-functional mechanism and characteristics are systematically investigated, aiming to achieve a low-frequency broadband dual-functional effect.

## 2. Theoretical Analysis

### 2.1. Structure Model

**Figure 1:**
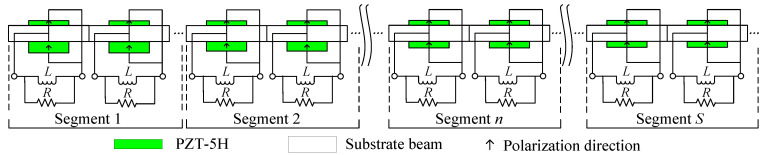
Schematic model of the gradient mode piezoelectric metamaterial beam.

The gradient mode piezoelectric metamaterial beam consists of *S* graded periodic segments. Each segment includes *j* periodic units with identical piezoelectric local oscillators, as illustrated in Figure 1. For simplicity, only two periodic units are illustrated in each segment. To achieve the asymmetric gradient distribution of the local oscillators, the thickness of the upper piezoelectric layer remains constant across all local oscillator units, while the thickness of the lower piezoelectric layer decreases gradually in a gradient manner along the length of the beam for each segment. This approach aims to broaden the dual-functional band and extend it towards lower frequency. To comprehensively investigate the impact of various gradient modes on the dual-functional properties, a variable *q* is introduced to denote the order of grading. The general formula for the graded thickness of the lower piezoelectric layer of the local oscillator in the *n*th segment can be expressed as

(1) $$h_{\left(3,n\right)}=h_{3}-\Delta h_{3}{\left(\frac{n-1}{S-1}\right)}^{q}\;n=1,\;2,\;\cdots S$$

where $h_{3}$ represents the thickness of the lower piezoelectric layer of the initial segmented local oscillator, $\Delta h_{3}$ denotes the variation range of the graded thickness, and *S* is the total number of segments.

**Figure 2:**
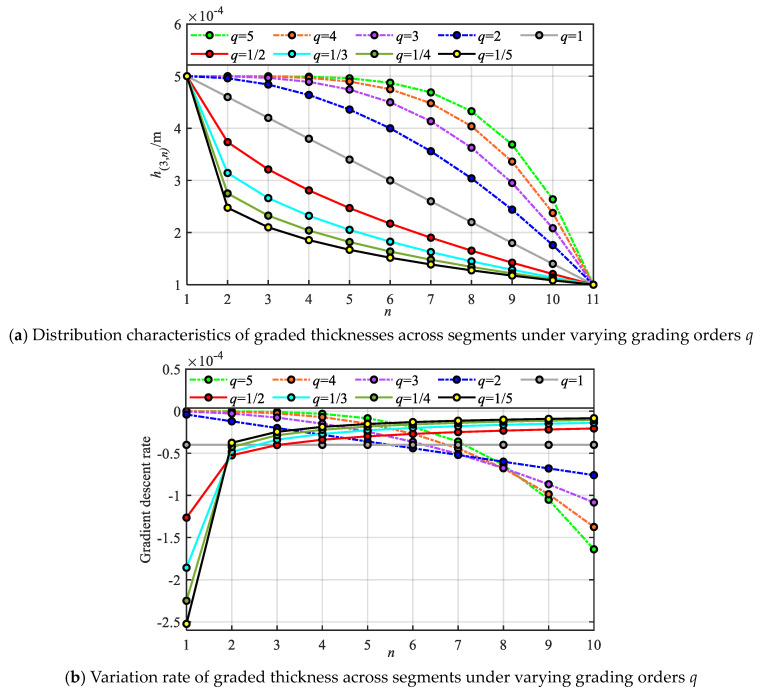
The influences law of gradient mode on the distribution and variation rate of grading parameter.

Here, the influence of various gradient modes on the construction of the metamaterial beam model is first investigated. The graded thickness is calculated using Equation (1), and the gradient distribution curves and gradient variation rates of the piezoelectric layer thickness of each local oscillator unit in different segments under various grading orders *q* are obtained, as illustrated in Figure 2, where $h_{3}=0.5\;\mathrm{mm}$, $\Delta h_{3}=0.4\;\mathrm{mm}$, S=11. It can be seen from Figure 2 that with the increase in the number of segments *n*, various gradient modes present different gradient change rules. Firstly, in the 1st-order mode, the graded thickness of each local oscillator unit in different segments shows a linear distribution, and the variation rate of graded thickness is constant $\Delta h_{3}$. In the fractional-order mode, the variation rate of graded thickness shows a gradually decreasing trend, that is, the graded thickness decreases faster near the initial segment and slower near the terminal segment, so the graded thickness in the fractional-order mode shows a distribution state of being sparse first and then dense along the length of the metamaterial beam; conversely, in the high-order mode, the graded thickness shows a distribution state of being dense first and then sparse along the length of the metamaterial beam. In addition, the graded thickness of the local oscillator units in the initial and terminal segments of all gradient modes is consistent.

### 2.2. The Propagation and Energy Harvesting Mechanism of Bending-Dominated Wave

**Figure 3:**
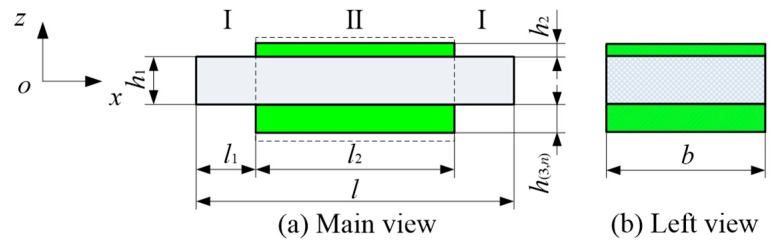
Schematic illustration of the *n*th segmented asymmetric local oscillator unit.

In the asymmetric mode, the coupling effect of waveforms manifests in the structure such that a single transverse excitation (or longitudinal excitation) not only induces a bending wave (or longitudinal wave) but also generates a corresponding longitudinal wave (or bending wave). For theoretical derivation, we consider the *n*th segment as an example. The structural schematic diagram of the local oscillator unit is illustrated in Figure 3 (shunt circuits are omitted). Here, I and II denote the regions occupied by the substrate beam and the piezoelectric layer, respectively.

Under the influence of the bending wave, the expression for the strain at any cross-section within the region occupied by the piezoelectric layer is:

(2) $$S(x,t)=\frac{\partial v(x,t)}{\partial x}+z\frac{\partial^{2}w(x,t)}{\partial x^{2}}$$

where $v(x,t)=V(x)e^{i\omega t}$ and $w(x,t)=W(x)e^{i\omega t}$ are longitudinal and transverse displacements, respectively.

It is evident from the precise charge integration method [36] that the total charge *Q* generated by the parallel piezoelectric bimorph can be expressed as:

(3) $$Q=\frac{d_{31}}{s_{11}^{E}}b\left[\frac{2h_{1}+h_{2}+h_{\left(3,n\right)}}{2}\left(\frac{\partial w(l_{2},t)}{\partial x}-\frac{\partial w(0,t)}{\partial x}\right)\right]\;+\varepsilon_{33}^{s}A_{s}({E^{\prime }}_{3}+{E^{\prime \prime }}_{3})\;$$

where ${E^{\prime }}_{3}$, ${E^{\prime \prime }}_{3}$ are the internal electric field strengths of the upper and lower piezoelectric layers, respectively; $s_{11}^{E}$ is the elastic compliance coefficient; $d_{31}$ is the strain constant; $\varepsilon_{33}^{s}$ is the constant strain dielectric constant; and $A_{s}$ represents the area of the piezoelectric electrode.

The additional stresses in the upper and lower piezoelectric layers at region II are further given as

(4) $$T_{ai}=\left\{\begin{array}{l} \frac{d_{31}^{2}sZb}{{\left(s_{11}^{E}\right)}^{2}h_{2}(1+sZ(C_{ps1}+C_{ps2}))}\left[\frac{2h_{1}+h_{2}+h_{\left(3,n\right)}}{2}\left(\frac{\partial w(l_{2})}{\partial x}-\frac{\partial w(0)}{\partial x}\right)\right]\;i=1\\ \frac{d_{31}^{2}sZb}{{\left(s_{11}^{E}\right)}^{2}h_{\left(3,n\right)}(1+sZ(C_{ps1}+C_{ps2}))}\left[\frac{2h_{1}+h_{2}+h_{\left(3,n\right)}}{2}\left(\frac{\partial w(l_{2})}{\partial x}-\frac{\partial w(0)}{\partial x}\right)\right]\;i=2 \end{array}\right.$$

where $C_{ps1}$ and $C_{ps2}$ are the intrinsic capacitances of the upper and lower piezoelectric layers, respectively; *Z* denotes the impedance of the shunt circuit; and *s* is the complex variable of Laplace transformation.

(5) $$C_{psi}=\left\{\begin{array}{l} \;\frac{\varepsilon_{33}^{s}A_{s}}{h_{2}}\;i=1\\ \frac{\varepsilon_{33}^{s}A_{s}}{h_{\left(3,n\right)}}\;i=2\; \end{array}\right.$$

Based on the Euler–Bernoulli beam theory, given that the asymmetry of the basic unit leads to the coupling between longitudinal and transverse vibrations, the corresponding coupled vibration equation can be expressed as [54]:

(6) $$\left\{\begin{array}{l} EA\frac{\partial^{2}v(x,t)}{\partial x^{2}}-\beta \frac{\partial^{3}w(x,t)}{\partial x^{3}}-\rho A\frac{\partial^{2}v(x,t)}{\partial t^{2}}+\alpha \frac{\partial^{3}w(x,t)}{\partial t^{2}\partial x}=0\\ EI\frac{\partial^{4}w(x,t)}{\partial x^{4}}-\beta \frac{\partial^{3}v(x,t)}{\partial x^{3}}+\rho A\frac{\partial^{2}w(x,t)}{\partial t^{2}}=0 \end{array}\right.$$

where

(7) $$\left\{\begin{array}{l} EA=E_{1}A_{1}+E_{2}A_{2}+E_{2}A_{3}\\ \rho A=\rho_{1}A_{1}+\rho_{2}A_{2}+\rho_{2}A_{3}\\ EI=E_{1}I_{1}+E_{2}I_{2}+E_{2}I_{3}\\ \alpha =\rho_{3}(A_{3}-A_{2})(h_{1}/2+h_{2}+\left(h_{\left(3,n\right)}-h_{2}\right)/2)\\ \beta =E_{3}(A_{3}-A_{2})(h_{1}/2+h_{2}+\left(h_{\left(3,n\right)}-h_{2}\right)/2) \end{array}\right.$$

where α and β represent the mass coupling coefficient and the stiffness coupling coefficient, respectively. EI, EA, and ρA represent the bending stiffness, tensile stiffness, and unit volume mass; $\rho_{1}$, $E_{1}$, $A_{1}$, and $I_{1}$ denote the density, Young’s modulus, cross-sectional area, and second moment of area of the substrate beam, while $\rho_{2}$, $E_{2}$, $A_{2}$, and $I_{2}$ correspond to the same properties for the upper piezoelectric layer. Similarly, $A_{3}$ and $I_{3}$ are the cross-sectional area and second moment of area of the lower piezoelectric layer.

Let $V(x)=Be^{\lambda x}$, $W(x)=Ce^{\lambda x}$, and substituting in Equation (6) to organize:

(8) $$a\lambda^{6}+b\lambda^{4}+c\lambda^{2}+d=0$$

where

(9) $$\begin{array}{l} a=EIEA-\beta^{2}\\ b=(EI\rho A-\alpha \beta )\omega^{2}\\ c=-EA\rho A\omega^{2}\\ d=-\rho^{2}A^{2}\omega^{4} \end{array}$$

The solution of Equation (8) is given by:

(10) $$\begin{array}{l} \lambda_{1,2}=\pm \sqrt{\sqrt[{3}]{-\frac{m}{2}+\sqrt{{\left(\frac{m}{2}\right)}^{2}+{\left(\frac{p}{3}\right)}^{3}}}+\sqrt[{3}]{-\frac{m}{2}-\sqrt{{\left(\frac{m}{2}\right)}^{2}+{\left(\frac{p}{3}\right)}^{3}}}-\frac{b}{3a}}\\ \lambda_{3,4}=\pm \sqrt{r\cdot \sqrt[{3}]{-\frac{m}{2}+\sqrt{{\left(\frac{m}{2}\right)}^{2}+{\left(\frac{p}{3}\right)}^{3}}}+r^{2}\cdot \sqrt[{3}]{-\frac{m}{2}-\sqrt{{\left(\frac{m}{2}\right)}^{2}+{\left(\frac{p}{3}\right)}^{3}}}-\frac{b}{3a}}\\ \lambda_{5,6}=\pm \sqrt{r^{2}\cdot \sqrt[{3}]{-\frac{m}{2}+\sqrt{{\left(\frac{m}{2}\right)}^{2}+{\left(\frac{p}{3}\right)}^{3}}}+r\cdot \sqrt[{3}]{-\frac{m}{2}-\sqrt{{\left(\frac{m}{2}\right)}^{2}+{\left(\frac{p}{3}\right)}^{3}}}-\frac{b}{3a}} \end{array}$$

where $p=\frac{c}{a}-\frac{b^{2}}{3a^{2}}$, $m=\frac{d}{a}+\frac{2b^{3}}{27a^{3}}-\frac{bc}{3a^{2}}$, $r=\frac{-1+\sqrt{3}i}{2}$.

Therefore, the displacement mode shape functions V(x) and W(x) can be formulated as:

(11) $$\begin{array}{l} W(x)=C_{1}e^{\lambda_{1}x}+C_{2}e^{\lambda_{2}x}+C_{3}e^{\lambda_{3}x}+C_{4}e^{\lambda_{4}x}+C_{5}e^{\lambda_{5}x}+C_{6}e^{\lambda_{6}x}\\ V(x)=B_{1}e^{\lambda_{1}x}+B_{2}e^{\lambda_{2}x}+B_{3}e^{\lambda_{3}x}+B_{4}e^{\lambda_{4}x}+B_{5}e^{\lambda_{5}x}+B_{6}e^{\lambda_{6}x} \end{array}$$

where

(12) $$B_{i}=H(\lambda_{i})C_{i}\;i=1,\;2,\;\cdots 6$$

where $H(\lambda_{i})=\frac{EI\lambda_{i}^{4}-\omega^{2}\rho A}{\beta \lambda_{i}^{3}}$.

The axial force F(x), turning angle θ(x), flexural moment M(x), and shear force Q(x) are, respectively:

(13) $$\begin{array}{l} F(x)=EA\frac{dV(x)}{dx}-\beta \frac{d^{2}W(x)}{dx^{2}}+\left(T_{a1}A_{2}-T_{a2}A_{3}\right)\\ \theta (x)=\frac{dW(x)}{dx}\\ M(x)=EI\frac{d^{2}W(x)}{dx^{2}}-\beta \frac{dV(x)}{dx}+\frac{h_{1}+h_{2}}{2}T_{a1}A_{2}+\frac{h_{1}+h_{\left(3,n\right)}}{2}T_{a2}A_{3}\\ Q(x)=EI\frac{d^{3}W(x)}{dx^{3}}-\beta \frac{d^{2}V(x)}{dx^{2}} \end{array}$$

The aforementioned mechanical quantities can be represented in the form of state vectors, i.e., φ=[V(x)F(x)W(x)θ(x) M(x)Q(x)]. In region I of the *n*th unit, the bending and the longitudinal waves are decoupled, simplifying the solution process. Therefore, a detailed explanation is not provided here.

From the transfer matrix method, the mechanical state vectors of the adjacent components at $x=nl,\;nl+l_{1},\;nl+l_{1}+l_{2}$ should remain continuous, and so there are:

(14) $$\psi_{n2}=T_{c}\psi_{(n-1)2}$$

where $T_{c}$ denotes the transfer matrix and $\psi_{n2}$ and $\psi_{(n-1)2}$ are expressed as:

(15) $$\begin{array}{l} \psi_{n2}=[C_{(n,2)1}\;C_{(n,2)2}\;C_{(n,2)3}\;C_{(n,2)4}\;C_{(n,2)5}\;C_{(n,2)6}]\\ \psi_{(n-1)2}=[C_{(n-1,2)1}\;C_{(n-1,2)2}\;C_{(n-1,2)3}\;C_{(n-1,2)4}\;C_{(n-1,2)5}\;C_{(n-1,2)6}] \end{array}$$

From Bloch’s theorem, we get:

(16) $$\left|T_{c}-e^{ikl}I\right|=0$$

For a given excitation frequency ω, the dispersion relation between the wave vector k and the frequency ω can be obtained by solving the eigenvalues of the transfer matrix $T_{c}$.

As illustrated in Figure 1, the local oscillator units between each segment of the gradient mode metamaterial beam structure do not conform to strict periodicity. Consequently, the bandgap characteristics of the overall structure cannot be directly described by the energy bands of conventional periodic systems, rendering the Bloch theorem inapplicable to the entire gradient mode metamaterial beam. However, each segment consists of *j* identical periodic units, allowing it to be treated as an effective periodic subsystem—referred to herein as a sub-metamaterial beam. By analyzing the energy band structures and group velocities of these sub-beams, the underlying mechanisms of bending-dominated wave propagation and energy localization within the gradient metamaterial beam can be effectively elucidated.

**Figure 4:**
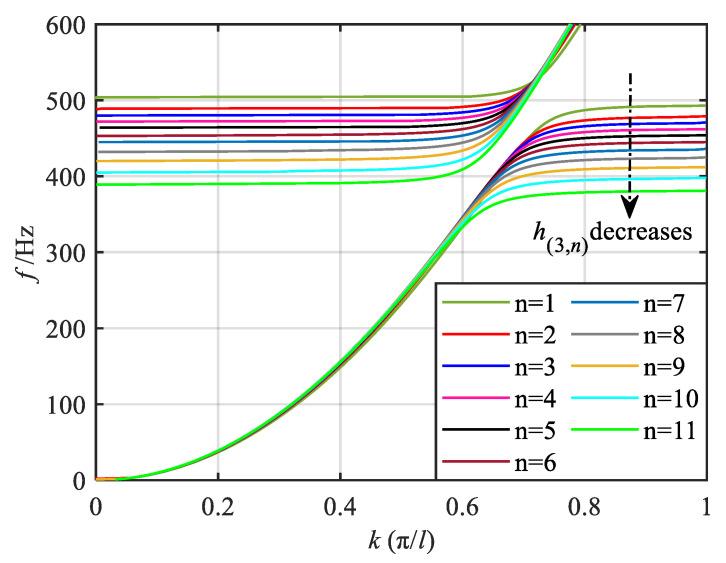
Energy band dispersion curves of bending-dominated wave for various segments.

**Figure 5:**
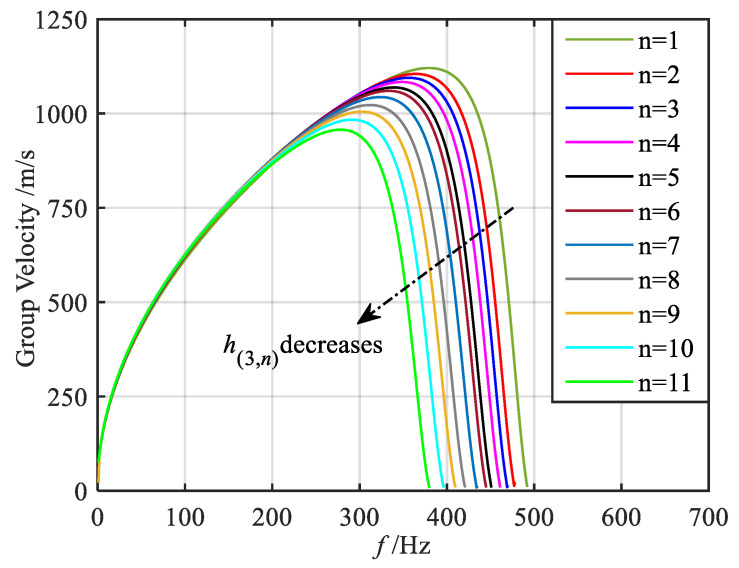
Group velocity curves of bending-dominated wave for various segments.

Here, taking the *q* = 1/2 order mode as an example, referring to Equation (16), the bending-dominated wave band structure of each segment of the metamaterial beam in the gradient mode can be calculated, as shown in Figure 4 and Figure 5, in which the band dispersion curves and the group velocity curves are given, respectively. It should be noted that, in the asymmetric mode, both the longitudinal and bending waves coexist within the structure. However, as evidenced by previous studies [50], under bending wave excitation, the bending deformation response is significantly more pronounced compared to the longitudinal deformation response, with a magnitude difference of approximately two orders of magnitude. Furthermore, the primary objective of this paper is to investigate the dual-functional broadening characteristics, with particular emphasis on elucidating the underlying mechanism of this dual-function broadening. The concept of wave coupling has been discussed in our previous works. Given the relatively weak longitudinal wave response under bending excitation, this paper does not focus extensively on the longitudinal wave mode. For clarity, therefore, only the band structure corresponding to the bending-dominated wave is presented in this paper. From the band dispersion curves of the 11 segments, it is evident that when the wave vector is small, all dispersion curves nearly overlap. As the wave vector increases, the dispersion curves gradually diverge and flatten. When the wave vector reaches the boundary of the Brillouin zone, the dispersion curves approach a flat band. The group velocity curves reveal that with increasing frequency, the group velocities of each segment initially increase to a peak and then decrease. At frequencies corresponding to the flat band of the dispersion curve, the group velocity approaches zero. Furthermore, Figure 4 and Figure 5 also indicate that as the graded thickness decreases, both the frequency corresponding to the flat band of the dispersion curve and the zero point of the group velocity curve decrease, implying that the segments with smaller graded thickness exhibit lower resonant frequencies.

Through the aforementioned analysis, it is evident that at the frequency level, the bending-dominated wave of varying frequencies attains its group velocity zero points in various segments. This results in distinct frequency positions of energy conversion. Specifically, lower frequency waves correspond to thinner graded thicknesses and consequently exhibit energy conversion at more posterior positions. Conversely, the higher frequency bending-dominated wave experiences energy conversion at more anterior positions. In summary, as the bending-dominated wave of different frequencies propagates along the direction of decreasing graded thickness, they cease forward propagation at varying segments, leading to the spatial frequency division phenomenon observed in these waves. At the wave vector level, the group velocity of the bending-dominated wave reaches zero at the boundaries of the Brillouin zone in each segment. This localization of energy at these positions results in enhanced wave field energy.

In conclusion, the bending-dominated wave propagating in the gradient mode metamaterial beam will form the rainbow trapping [55,56,57,58] effect described above, which is conducive to the widening of the bandgap range and the energy harvesting band. Subsequently, the dual-functional properties of vibration reduction and energy harvesting in gradient mode metamaterial beam will be analyzed.

### 2.3. Vibration Reduction Performance Analysis

#### 2.3.1. Evaluation Criteria and Vibration Reduction Properties of Various Gradient Modes

Given that the gradient mode metamaterial beam is an aperiodic structure and the propagation properties of the bending-dominated wave cannot be directly characterized by the band structure, this study investigates its vibration reduction performance using the vibration transmission properties of the finite structure. The corresponding structural, material, and circuit parameters are detailed in Table 1. In the analysis, the right end of the beam is set as a free boundary condition, while a transverse harmonic displacement excitation with amplitude $W_{in}$ and frequency ranging from 350 Hz to 525 Hz is applied at the left end. The excitation acceleration $a_{cc}=\omega^{2}W_{in}$ is maintained at a constant value of 10 g. Under these boundary conditions, the transverse displacement response amplitude $W_{out}$ at the right end of the beam can be determined using the transfer matrix method, from which the bending vibration transmission rate τ is subsequently calculated. The formula for calculating τ is as follows:

(17) $$\tau (f)=20\mathrm{lg}\left(\frac{W_{out}}{W_{in}}\right)$$

To facilitate the discussion of the vibration suppression performance of the metamaterial dual-functional beam under various gradient modes, the attenuation bandwidth Δf and the attenuation area Ω (the area surrounded by the attenuation region of the transmission rate curve and the zero line) of the vibration transmission properties are adopted as evaluation indexes for quantitative analysis in this section. The calculation formulas are as follows:

(18) $$\left\{\begin{array}{l} \Delta f=\sum_{i=1}^{t}\left(f_{ci}-f_{si}\right)\;t=1,\;2,\;3\cdots \\ \Omega =\sum_{i=1}^{t}\int_{f_{si}}^{f_{ci}}\left|\tau (f)\right|df\;(\tau (f)<0) \end{array}\right.$$

where *t* is the number of segments in the attenuation region, and $f_{ci}$ and $f_{si}$ are the cut-off and starting frequencies of the *i*th attenuation band, respectively.

**Figure 6:**
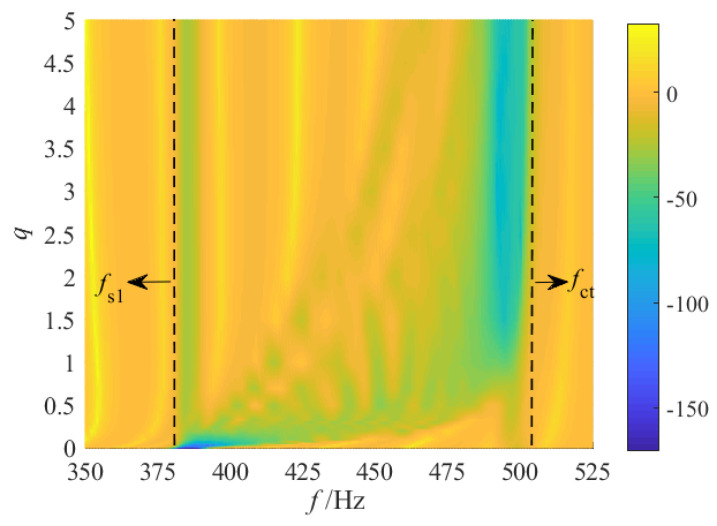
Vibration transmittance properties for various gradient modes.

The numerical calculations are performed using the transfer matrix method, resulting in distribution cloud maps of vibration transmission properties under various gradient modes, as illustrated in Figure 6. From these cloud maps, it is evident that various gradient modes lead to significant variations in both the attenuation bandwidth and the maximum attenuation level.

For the fractional-order mode, when 0 < *q* ≤ 1/2, as *q* increases, the cut-off frequency of the attenuation region progressively shifts towards higher frequencies, leading to a substantial increase in the attenuation bandwidth. However, when 1/2 < *q* < 1, although the attenuation region continues to extend into the high-frequency band, the low-frequency band of the attenuation region gradually contracts, causing the overall attenuation bandwidth to decrease. The maximum attenuation bandwidth boundaries for the entire fractional-order mode are indicated by the dotted lines labeled $f_{s1}$ and $f_{ct}$ in Figure 6. According to the analysis in Section 2.1, the graded thickness in the fractional-order mode exhibits a rapid drop in the initial segment and follows a distribution characteristic that is initially sparse and subsequently dense. Consequently, when *q* is small, the significant changes in graded thickness within the first few segments result in large differences between adjacent resonant frequencies, making it difficult for adjacent resonant bandgaps to couple effectively and leading to discontinuities in the latter part of the attenuation region. As *q* increases, the changes in graded thickness near the starting position diminish while those near the termination position increase, resulting in a continuous attenuation region in the latter half but potential discontinuities in the first half.

Secondly, for the 1st-order linear grading mode, similar to the fractional-order case approaching the 1st-order, a broadband attenuation effect can also be achieved. Notably, the entire attenuation region is discontinuous. The initial region is partitioned into several distinct attenuation regions by multiple resonance peaks, whereas the latter remains relatively continuous. Additionally, the maximum attenuation within the entire attenuation region is enhanced compared to the fractional-order.

For the high-order grading mode, as *q* increases, the overall distribution of the attenuation region progressively shifts toward $f_{ct}$. The attenuation region in the low-frequency band diminishes further, and the attenuation bandwidth gradually narrows. However, the maximum attenuation within the bandgap increases incrementally. This phenomenon occurs because, under the high-order mode, the graded thickness decreases slowly at the initial segments, exhibiting a distribution pattern that is initially dense and subsequently sparse. As *q* increases, the graded thickness of the first few segments becomes increasingly dense, leading to a smaller frequency spacing between adjacent resonances. Consequently, the adjacent resonance bandgaps are effectively coupled, resulting in enhanced attenuation in the latter part of the attenuation region. Meanwhile, the variation in graded thickness between segments near the termination position gradually widens, leading to larger differences in adjacent resonance frequencies. As a result, a noticeable discontinuity emerges in the earlier portion of the entire attenuation region.

Finally, since the graded thicknesses of the initial and terminal segments of the metamaterial beam remain constant across all gradient modes, corresponding to identical resonant frequencies, the frequency bands near the dotted line position (indicating the boundary of the maximum attenuation bandwidth) consistently exhibit specific vibration attenuation properties regardless of the chosen thickness grading mode. Consequently, the attenuation bandwidth Δf of the vibration attenuation region is influenced not only by the grading mode but also by the distribution of resonant frequencies in the initial and terminal segments.

**Figure 7:**
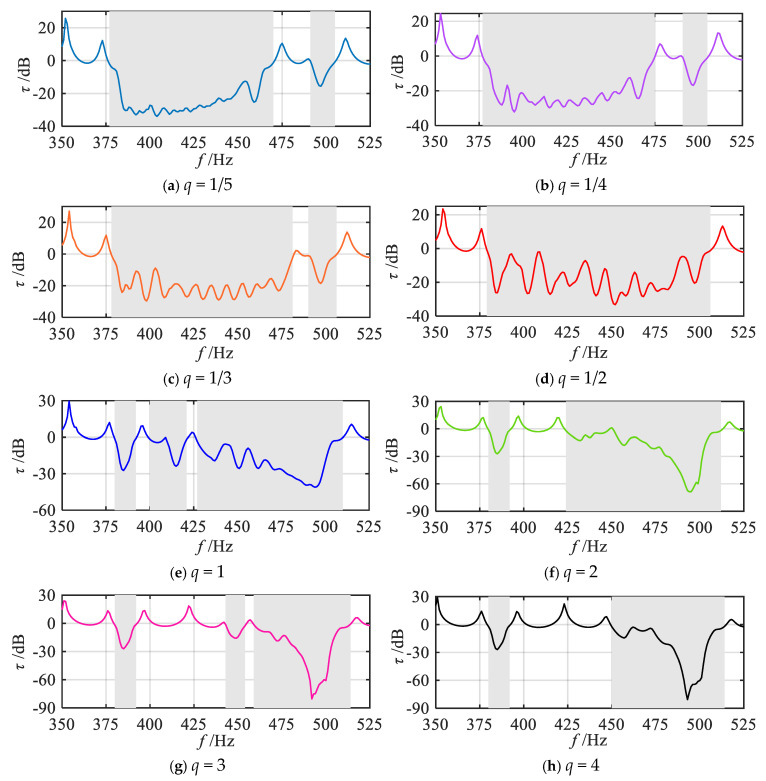

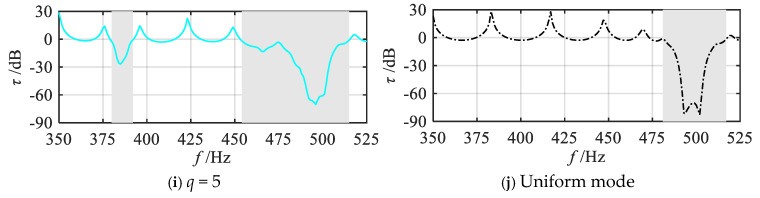
Slices of vibration transmittance properties for various gradient modes.

**Figure 8:**
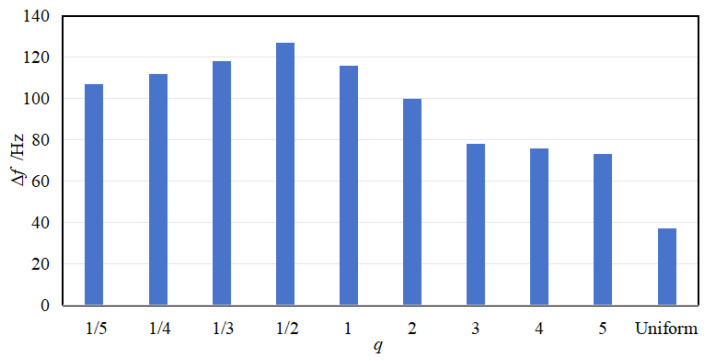
Attenuation bandwidth for various gradient modes.

**Figure 9:**
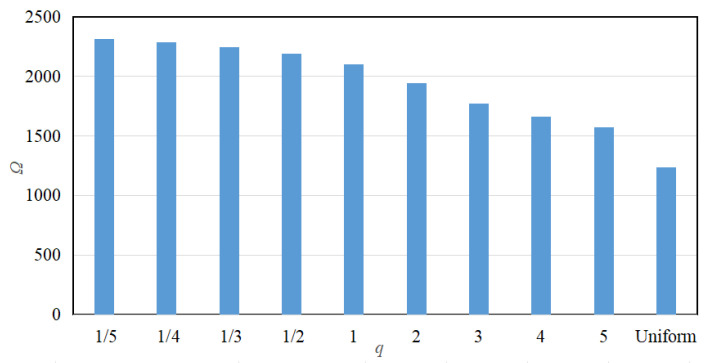
Attenuation area for various gradient modes.

To further investigate the effects of various gradient modes on vibration reduction properties, a quantitative analysis of the attenuation bandwidth and attenuation area under various grading orders *q* is conducted. Corresponding to Figure 6, slice curves of vibration transmission properties for several grading orders *q* are extracted, with results presented in Figure 7. The *q* values include 1/5, 1/4, 1/3, 1/2, 1, 2, 3, 4, and 5. For ease of comparison, Figure 7j also shows the frequency response curve of vibration transmission properties in the uniform mode (i.e., where all segmented units have the same thickness as the initial unit). First, the positions of the attenuation regions for each gradient mode are identified based on Figure 7, as indicated by the shaded areas. Through comparison, it can be observed that in the high-order mode, the attenuation within the frequency range of 450–475 Hz is minimal. This phenomenon primarily occurs because, in the high-order mode, the graded thickness follows a distribution pattern that is initially dense and subsequently sparse. Consequently, the span between resonance frequencies near the termination segment becomes relatively large. Taking Figure 7i as an example, the resonance frequencies of the 9th and 10th segments are 483 Hz and 463 Hz, respectively. As a result, the bandgaps of adjacent segments fail to couple effectively, and the period number *j* of the same unit within each segment remains relatively low, leading to reduced attenuation. Subsequently, the attenuation bandwidth and attenuation area for each gradient mode are calculated using Equation (18), with the results shown in Figure 8 and Figure 9.

The results show that the attenuation bandwidth and attenuation area exhibit different patterns of change. With the increase in *q*, the attenuation bandwidth exhibits the trend of increasing and then decreasing, and the maximum attenuation bandwidth is reached at the order of *q* = 1/2. Conversely, the attenuation area exhibits a monotonically decreasing trend, i.e., the attenuation area is the largest in fractional-order, the following in 1st-order, and the smallest in high-order. Further, the comparison also shows that the values of attenuation bandwidth and attenuation area are the smallest in the uniform mode.

#### 2.3.2. Effects of the Grading Parameters on Vibration Reduction

**Figure 10:**
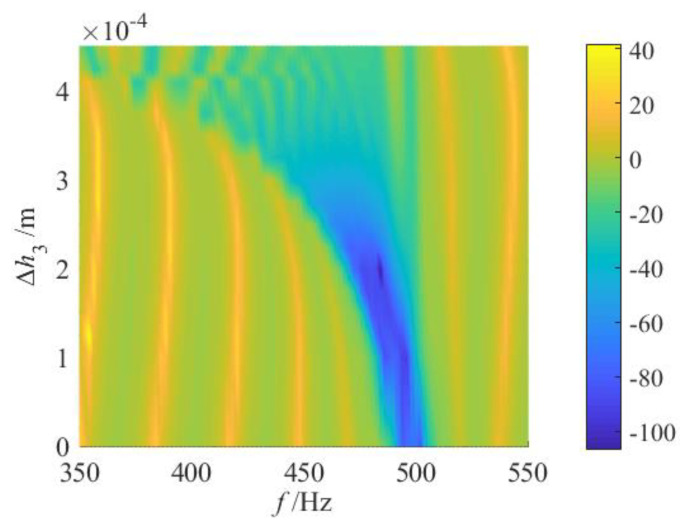
Vibration transmittance properties for various $\Delta h_{3}$.

The attenuation bandwidth of the vibration attenuation region is not only influenced by the gradient mode but also closely associated with the resonant frequencies of the initial and terminal segments. Given that the initial segment’s value is fixed, the graded thickness of the terminal segment is primarily determined by the variation range $\Delta h_{3}$ of the graded thickness. Consequently, the impact of $\Delta h_{3}$ on the transmission properties of the bending-dominated wave will be further explored below. Considering the superior vibration suppression performance demonstrated by fractional-order systems, the case where *q* = 1/2 is analyzed as an example. Let the $\Delta h_{3}$ range be 0~0.45 mm, where $\Delta h_{3}$ = 0 corresponds to the uniform mode. Figure 10 illustrates the transmission properties of bending-dominated waves for various $\Delta h_{3}$.

It can be observed that the attenuation bandwidth is minimal when $\Delta h_{3}$ = 0. As $\Delta h_{3}$ increases, the attenuation bandwidth expands significantly, and the starting frequency of the attenuation region progressively shifts toward lower frequencies, while the cut-off frequency remains largely unchanged. When $\Delta h_{3}$ reaches a certain threshold (e.g., $\Delta h_{3}$ = 0.425 mm), the attenuation region transitions from a continuous state to a discrete state. This phenomenon arises because the number of segments *S* remains constant. An increase in $\Delta h_{3}$ results in a greater disparity in graded thickness between adjacent segments, thereby amplifying the span of resonant frequencies of the local oscillator. Consequently, the coupling between adjacent resonant bandgaps weakens, leading to the discontinuity of the attenuation region.

**Figure 11:**
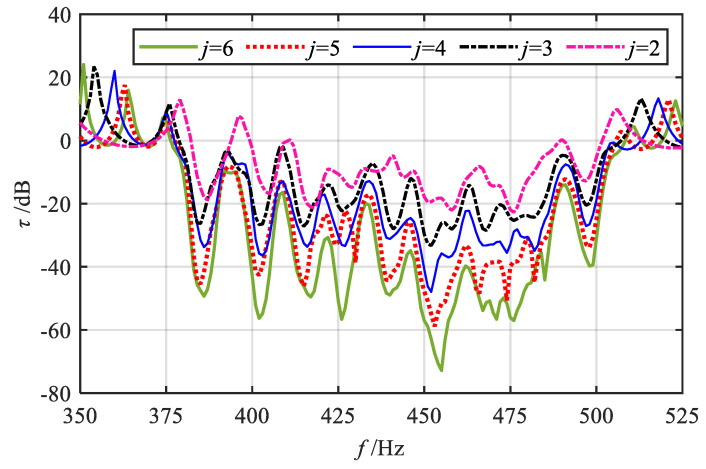
Effect of the number of period *j* of the same unit within each segment on the bending vibration transmittance properties.

Finally, it is worthwhile to investigate the influence of the number of periods *j* of the same unit within each segment on the vibration transmission properties. Taking the fractional-order mode (*q* = 1/2) as an example and setting $\Delta h_{3}$ = 0.4 mm, the range of *j* is defined from 2 to 6. The transmission curves are illustrated in Figure 11. The results indicate that as the number of period units *j* increases, the overall profile of the bending vibration transmission curve remains largely unchanged. However, the attenuation intensity at each resonant frequency and the overall attenuation region are significantly enhanced. Moreover, some resonance peaks near the attenuation zone are effectively suppressed, leading to a noticeable increase in the attenuation bandwidth. Consequently, by modulating the number of periods *j* of the same unit within each segment, the vibration attenuation performance of the gradient mode metamaterial beam can be efficiently controlled, achieving strong and broadband attenuation of vibration waves.

### 2.4. Energy Harvesting Performance Analysis

Take the *j*th power generation unit in the *n*th segment as an example. Based on Kirchhoff’s current law, it can be deduced that:

(19) $$(C_{ps1}+C_{\left(ps2,n\right)})v^{\prime }(t)+\frac{v(t)}{Z}-\vartheta \left[\frac{\partial^{2}w_{n2,j}(l_{2},t)}{\partial x\partial t}-\frac{\partial^{2}w_{n2,j}(0,t)}{\partial x\partial t}\right]=0$$

where

(20) $$\vartheta =\frac{d_{31}b(2h_{1}+h_{2}+h_{\left(3,n\right)})}{2s_{11}^{E}}$$

represents the electromechanical coupling coefficient of the dual-functional system.

After applying the Laplace transform, the expression for the output voltage of the *j*th piezoelectric bimorph in the *n*th segment can be derived as follows:

(21) $$V_{n,j}=\frac{\vartheta s}{\left(1/Z+s(C_{ps1}+C_{\left(ps2,n\right)})\right)}\left[\frac{\partial W_{n2,j}(l_{2})}{\partial x}-\frac{\partial W_{n2,j}(0)}{\partial x}\right]$$

It can therefore be deduced that the output power of the piezoelectric bimorph is:

(22) $$P_{n,j}=\frac{V_{n,j}^{2}}{2R}=\frac{{\left(\frac{\vartheta s}{\left(1/Z+s(C_{ps1}+C_{\left(ps2,n\right)})\right)}\left[\frac{\partial W_{n2,j}(l_{2})}{\partial x}-\frac{\partial W_{n2,j}(0)}{\partial x}\right]\right)}^{2}}{2R}$$

Since the majority of the energy is absorbed by the 1st oscillator at the resonant frequency, by aggregating the output power of the 1st oscillator across each segment, an approximate value of the total output power *P* for each segment can be obtained, which is expressed as follows:

(23) $$P=\sum_{n=1}^{S}\frac{{\left(\frac{\vartheta s}{\left(1/Z+s(C_{ps1}+C_{\left(ps2,n\right)})\right)}\left[\frac{\partial W_{n2,1}(l_{2})}{\partial x}-\frac{\partial W_{n2,1}(0)}{\partial x}\right]\right)}^{2}}{2R}$$

Since the research goal of this paper is to simultaneously achieve the dual-functional properties of vibration reduction and energy harvesting, the bands that can satisfy this condition are primarily the local resonant bandgap and the passbands near the band-edge. However, in the gradient mode, the segments of the metamaterial beam structure lack periodicity, making it impossible to directly obtain the bandgap and band-edge structure of the metamaterial through the Bloch’s theorem. Therefore, based on the attenuation regions of vibration waves in each gradient modes, the energy harvesting performance within these attenuation regions is mainly analyzed.

To facilitate the discussion of the energy harvesting performance of metamaterial beam in various gradient modes, this section adopts two evaluation indexes for quantitative analysis: the energy harvesting bandwidth Δf (consistent with the attenuation bandwidth) and the energy harvesting area Θ (the area enclosed by the output power curve and the zero line within the bandwidth range). The corresponding expressions are as follows:

(24) $$\left\{\begin{array}{l} \Delta f=\sum_{i=1}^{t}\left(f_{ci}-f_{si}\right)\;t=1,\;2,\;3\ldots \\ \Theta =\sum_{i=1}^{t}\int_{f_{si}}^{f_{ci}}P(f)df \end{array}\right.$$

where *t* represents the number of segments in the energy harvesting region, $f_{ci}$ and $f_{si}$ denote the cut-off and starting frequencies of the *i*th energy harvesting band, respectively.

**Figure 12:**
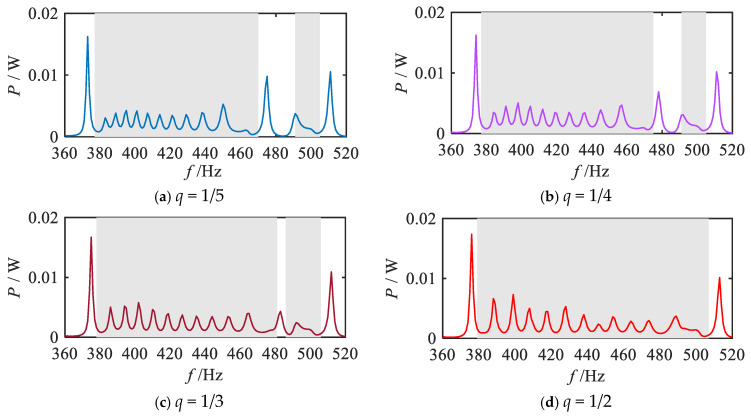

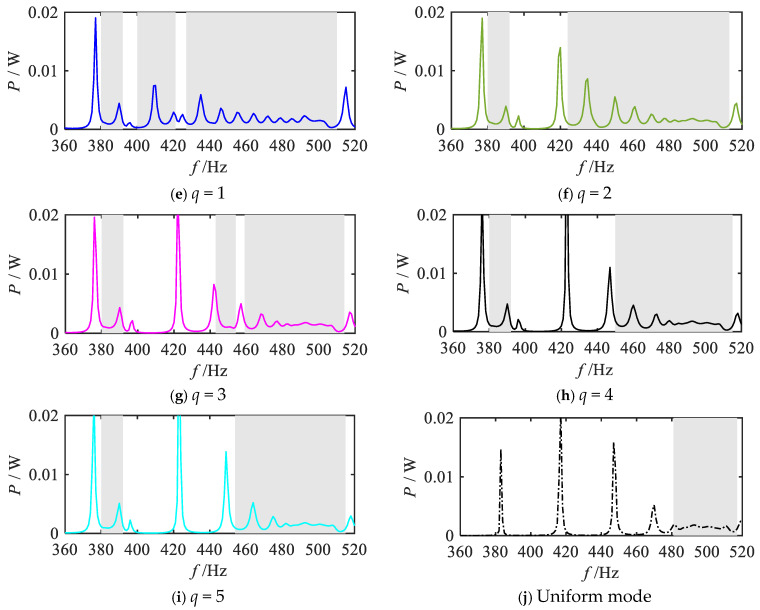
Frequency response curves of the total output power for various gradient mode.

For different values of *q* (1/5, 1/4, 1/3, 1/2, 1, 2, 3, 4, 5), the total output power calculation is carried out according to Equation (23), and the corresponding total output power frequency response curves are obtained, and the results are shown in Figure 12, where the shaded area indicates the attenuation region of the bending vibration. For comparison, the total output power frequency response curve in the uniform mode is also given in Figure 12.

**Figure 13:**
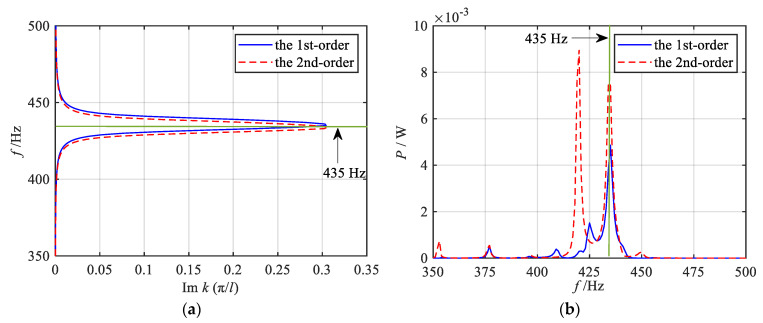
Energy band curves and output power curves of the 9th segment in the 1st-order mode and the 10th segment in the 2nd-order mode. (**a**) Energy band curves; (**b**) Output power curves.

Firstly, the energy harvesting properties within the attenuation regions under each grading mode are systematically investigated. The analysis of vibration reduction properties revealed that the fractional-order modes exhibit a broader attenuation region for bending vibration compared to other modes. Consequently, a significant and continuous broadband energy harvesting effect is observed within the bending vibration attenuation region (shadow region), as illustrated in Figure 12a–d. As the parameter *q* increases, the energy harvesting bandwidth expands gradually, reaching its maximum when *q* = 1/2. Similarly, the 1st-order mode also demonstrates the broadband energy harvesting capability, as shown in Figure 12e. Figure 12f–i depict the total output power frequency response curves under high-order modes, indicating that a broadband energy harvesting effect still exists in the attenuation region of the vibration wave. However, compared with the fractional-order and 1st-order modes, the bandwidth of the energy harvesting region is notably reduced, and the overall energy harvesting band is shifted backward. In addition, by comparing the output power curves of each order mode, it can be observed that distinct power peaks (e.g., at 435 Hz) appear in the shadow attenuation regions of the 1st-order and 2nd-order modes, as shown in Figure 12e–f, where these peaks are more pronounced compared to other sub-figures in Figure 12. This phenomenon is primarily attributed to the distribution of specific structural parameters. As illustrated in Figure 2, the graded thicknesses of the 9th segment in the 1st-order mode and the 10th segment in the 2nd-order mode are nearly identical (0.18 mm and 0.176 mm, respectively), resulting in closely matched resonant frequencies. Consequently, further analyses of the energy bands and output powers of these two segments are conducted, as presented in Figure 13. The results indicate that, for the 1st-order mode, 435 Hz falls within the bandgap frequency range of the 9th segment; similarly, in the 2nd-order mode, 435 Hz lies within the bandgap frequency range of the 10th segment. According to previous studies, piezoelectric metamaterials demonstrate effective energy harvesting performance within the bandgap frequency range, with power peaks typically occurring near the resonant frequency. Therefore, both the 1st-order and 2nd-order modes exhibit relatively high output power peaks at 435 Hz. Figure 12j presents the total output power frequency response curve under the uniform mode. Despite exhibiting energy harvesting performance in the bending vibration attenuation region, the uniform mode suffers from a narrower attenuation bandwidth, which consequently limits the energy harvesting interval.

**Figure 14:**
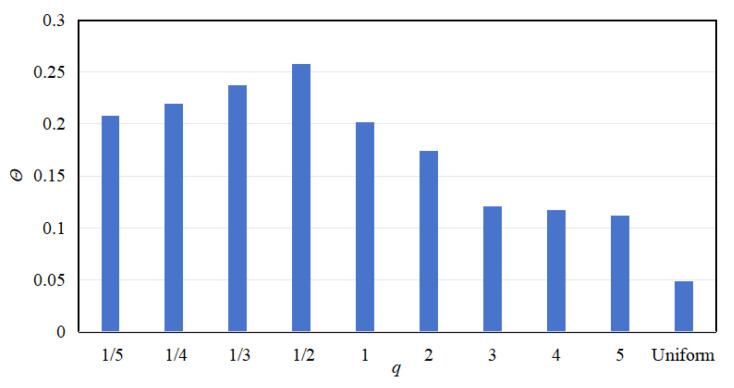
Energy harvesting area for various gradient modes.

Secondly, a comparative analysis is performed on the energy harvesting area within the energy harvesting region under each gradient mode. The energy harvesting areas under various gradient modes are calculated using Equation (24), and the results are presented in Figure 14. It can be observed that the energy harvesting area is larger in the fractional-order and 1st-order modes, but decreases in higher-order modes, reaching its maximum at the *q* = 1/2 order. The energy harvesting area within the uniform mode is also given in Figure 14, which is smaller due to the narrower attenuation bandwidth in the uniform mode. These findings suggest that there is a positive correlation between the energy harvesting bandwidth and the energy harvesting area, where a wider bandwidth generally corresponds to a larger energy harvesting area.

**Figure 15:**
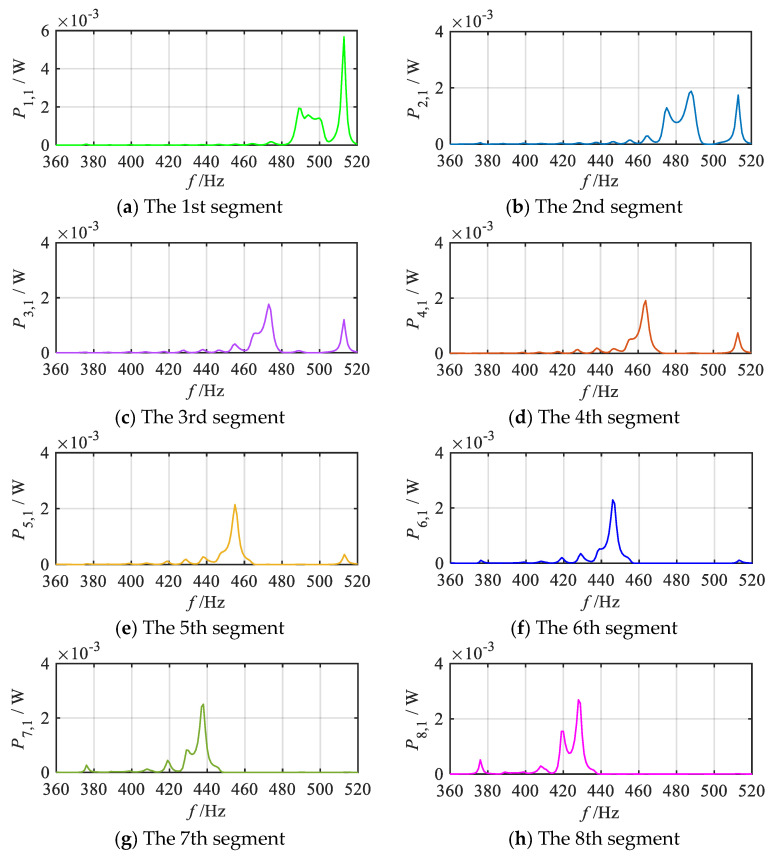

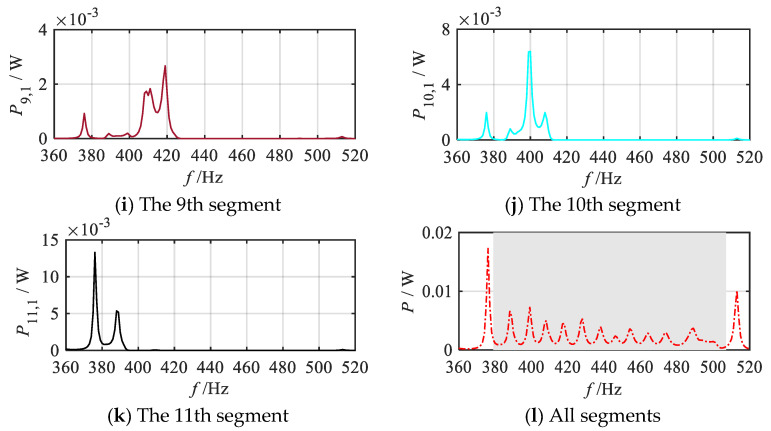
Frequency response curves of output power for the 1st oscillators within each segment and total output power of the gradient metamaterial beam (*q* = 1/2 order mode).

Finally, for enhanced clarity, the energy harvesting performance of each segment in the *q* = 1/2 order mode is further analyzed. The primary focus is on the output power of the 1st oscillator of each segmented unit, with the results presented in Figure 15. As illustrated in Figure 15a–k, the 1st oscillators of each segment demonstrates excellent energy harvesting performance near the resonant frequency of the corresponding local oscillator of the sub-metamaterial beam. Specifically, the energy harvesting bands of the 1st oscillators for segments 1~11 are (482 Hz~507 Hz), (469 Hz~494 Hz), (460 Hz~481 Hz), (451 Hz~472 Hz), (444 Hz~464 Hz), (434 Hz~455 Hz), (424 Hz~448 Hz), (412 Hz~437 Hz), (401 Hz~424 Hz), (383 Hz~411 Hz), (379 Hz~394 Hz), respectively. The energy harvesting bands vary across segments, with the highest band observed in the 1st segment and a gradual decrease in subsequent segments. This trend suggests that the position of the enhanced wave field energy follows a gradient variation law. The overlapping of the energy harvesting bands across segments results in a continuous broadband energy harvesting effect, as reflected in the total output power frequency response curve.

## 3. Finite Element Method

To further illustrate the reliability of the theoretical analysis, this section conducts a finite element simulation of the dual-functional performance of gradient mode metamaterial beams using COMSOL Multiphysics 5.4, a multi-physics coupling software platform. The simulation models for each grading order of the metamaterial beam are established with identical structural configurations, material properties, and circuit component parameters as those used in the theoretical analysis. A systematic comparison between the simulation results and theoretical predictions is subsequently performed to assess the consistency and accuracy of the analytical framework. The simulation environment settings are shown in Table 2.

**Figure 16:**
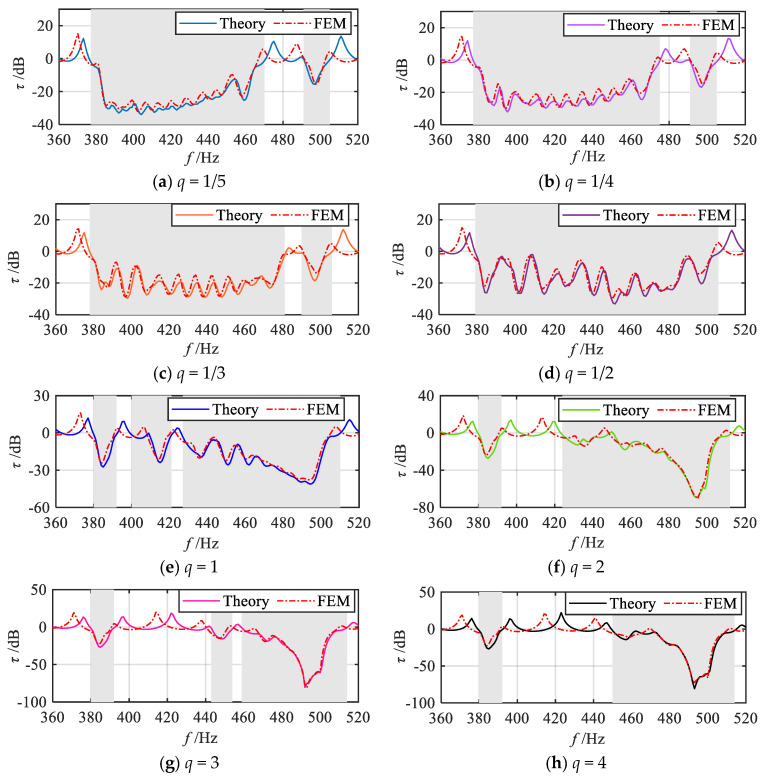

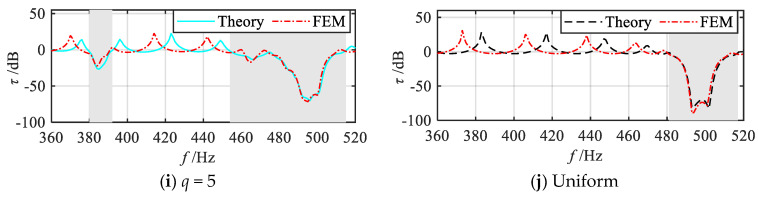
Simulation and theoretical results of vibration transmission properties under various gradient modes.

Firstly, finite element simulations are performed to analyze the vibration attenuation properties. Figure 16 presents the finite element simulation results and theoretical analysis results for the frequency response curves of bending vibration transmission properties under various grading modes *q* (1/5, 1/4, 1/3, 1/2, 1, 2, 3, 4, 5). The shaded regions indicate that the theoretically predicted attenuation frequency bands for each grading mode. As shown in Figure 16, the vibration transmission curves obtained from the finite element simulations exhibit a certain degree of attenuation within these shaded frequency bands. Upon further comparison, it can be observed that, aside from minor discrepancies in the positions of some peaks and valleys, the simulation results align well with the theoretical predictions in terms of overall trend. However, more noticeable differences are observed within the passbands (non-shaded regions), the resonance peak frequencies in the finite element results are lower than those predicted by the theoretical model. This discrepancy primarily arises from differences between the simulation and theoretical models. The theoretical model neglects the effects of shear deformation and the moment of inertia of the cross-section about the neutral axis, whereas these factors are incorporated into the finite element simulation. In reality, the shear deformation reduces beam stiffness, while the moment of inertia increases beam inertia, both of which contribute to a reduction in the structure’s natural frequency. Therefore, it is reasonable to observe that the resonance peak frequencies in the finite element results are lower than those in the theoretical results.

**Figure 17:**
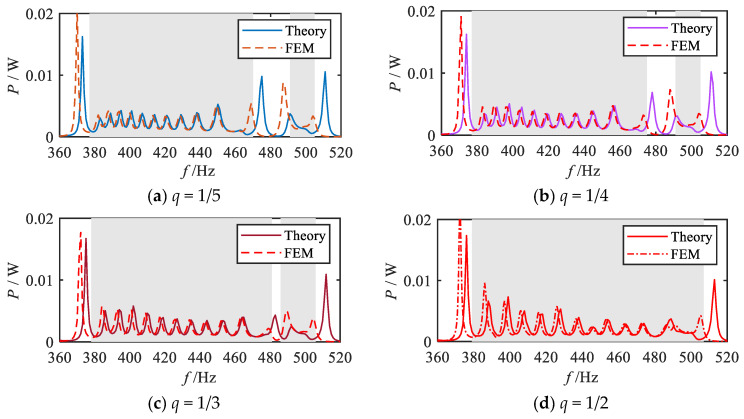

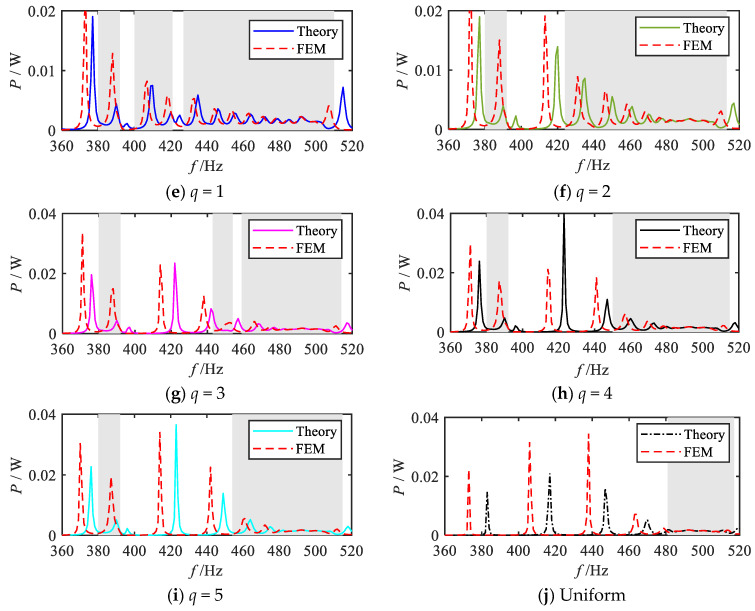
Comparison between simulation analysis results and theoretical analysis results for the total output power frequency response curves under various grading modes.

Furthermore, finite element simulations are performed to evaluate the energy harvesting performance of the metamaterial beam. Figure 17 presents the finite element results and theoretical analysis results of the total output power frequency response curves under each grading mode *q* (1/5, 1/4, 1/3, 1/2, 1, 2, 3, 4, 5). The shaded regions indicate the theoretically predicted attenuation frequency bands for each grading mode. It can be observed that within these shaded frequency bands, the simulated output power curves for all grading modes exhibit a continuous broadband energy harvesting effect. Furthermore, by comparing the theoretical predictions with the simulation results, it is evident that the trends of the output power frequency response curves within the shaded bands align closely with the theoretical analysis. In contrast, in the non-shaded frequency bands, noticeable discrepancies between the theoretical and simulation results are observed, particularly in the positions of the power peaks. The peak frequencies in the finite element results are lower than those predicted theoretically, which corresponds to the peak shift observed in the vibration transmission simulation curves. The observed deviation in the output power curves can be attributed to the reduced natural frequency of the metamaterial beam structure in the simulation environment.

**Figure 18:**
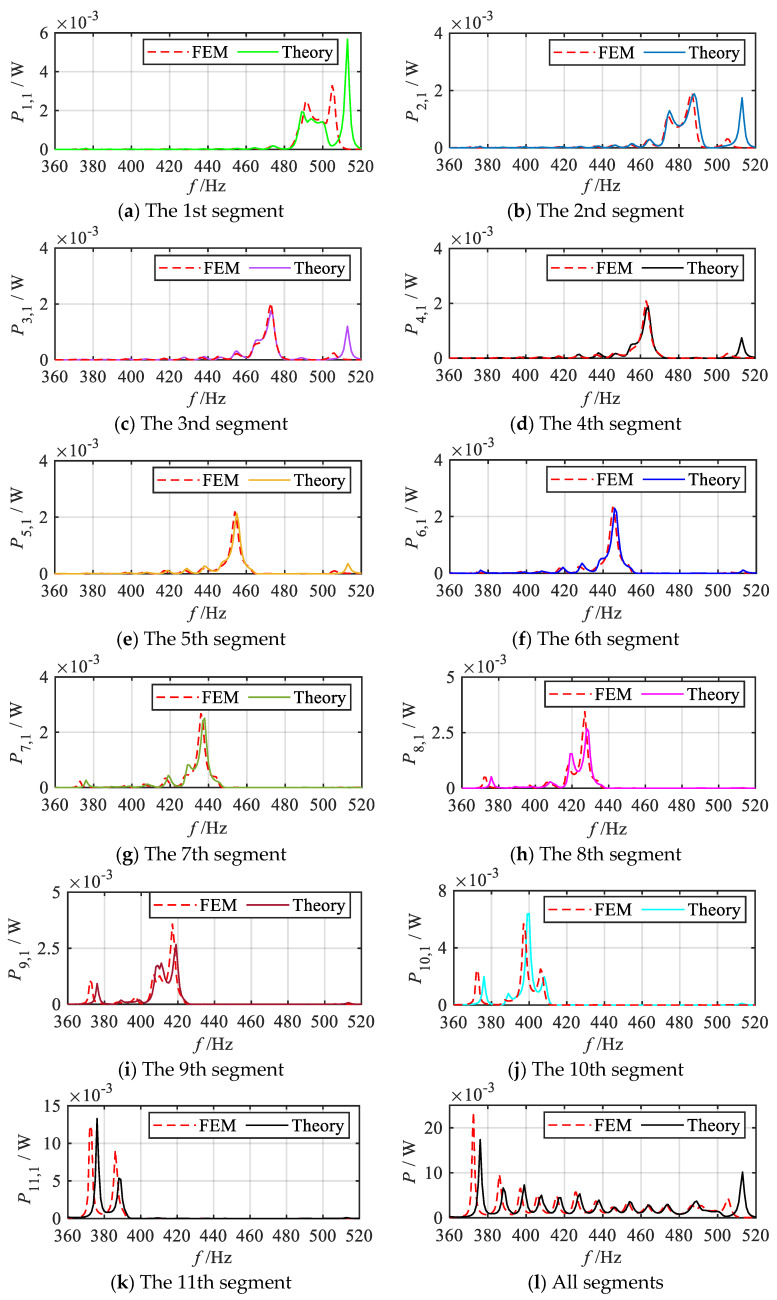
Simulation and theoretical results of frequency response curves of output power for the 1st oscillator within each segment and total output power of the gradient metamaterial beam (*q* = 1/2 order mode).

The energy harvesting properties of each segmental unit in the gradient mode metamaterial beam are further analyzed and numerically evaluated using the finite element method. Consistent with the theoretical analysis, taking the *q* = 1/2 order mode as an example, Figure 18 presents the simulation results of the output power frequency response curves of the 1st oscillators of each segment under the *q* = 1/2 order mode. For ease of comparison, the theoretical analysis results are also included in the figure. As shown in the simulation results, the 1st oscillators of the 1st to 11th segments all demonstrate effective energy harvesting performance near their respective resonance frequencies. Moreover, the energy harvesting frequency bands across these segments exhibit a gradual shift from medium-high frequencies to low frequencies. Since the energy harvesting frequency bands of the adjacent segments partially overlap, a continuous broadband energy harvesting effect is clearly reflected in the total output power frequency response curve, as illustrated in Figure 18l. Furthermore, a detailed comparison between the theoretical and simulation curves reveals the slight discrepancies in the output power values and peak positions. Nevertheless, the overall trends of the frequency response curves remain highly consistent.

## 4. Conclusions

In this paper, based on the idea of grading, a gradient mode piezoelectric metamaterial beam structure is constructed, and a broadband study of the dual-functional properties is carried out. Firstly, the influence of various gradient modes on the grading parameters and grading variation rate of each segment of the metamaterial beam is investigated; then, the band structure and group velocity of each segment are studied, and the propagation mechanism of the bending-dominated wave and the energy harvesting mechanism are elucidated. Furthermore, the evaluation criteria of the dual-functional properties in the gradient mode are proposed, and the broadening laws of the dual-functional band under various gradient modes are discussed. Finally, finite element simulation is conducted to analyze and verify the theoretical results. The study reveals that:

(1) In the gradient mode metamaterial beam, the propagation of the bending waves leads to spatial frequency division and enhanced wavefield energy, enabling each segment to efficiently capture bending wave energy at its corresponding resonant frequency, thereby significantly improving energy conversion efficiency.
(2) Compared with the uniform mode metamaterial beam, the dual-functional properties bandwidth of the gradient mode metamaterial beam is significantly improved with the same electrical parameters, and the drawback of the existing graded method, which needs to use a large inductance element to realize the low frequency, is also avoided. The gradient mode and the variation range of graded thickness have a significant effect on the dual-functional performance, and optimal performance can be achieved by appropriately selecting these parameters.

Although this study has achieved the aforementioned research outcomes and innovations, certain limitations remain. Notably, further experimental validation has not yet been conducted. As an initial theoretical investigation, the primary objective and core contribution of this work lie in the proposal of a novel theoretical framework, which aims to establish the theoretical feasibility and potential significance of broadband dual-functional properties, thereby offering a foundational basis and directional guidance for future experimental studies. With respect to the relationship between theory and experiment, a strong qualitative consistency is anticipated between the proposed model and expected experimental observations. The model reliably predicted the evolving trends of dual-functional characteristic curves across various grading modes. However, with regard to quantitative precision, certain simplifying assumptions inherent in the theoretical framework (such as the neglect of shear deformation, rotational inertia, and structural damping) may result in discrepancies between predicted absolute values and actual experimental measurements. Consequently, this theoretical study offers a clear physical understanding and qualitative guidance for subsequent experimental investigations.

## Figures and Tables

**Table 1:** Structural, material, and circuit parameters of the gradient mode metamaterial beam.

Items	Parameters	Values	Units
PMMA	$\rho_{1}$	1142	kg/m^3^
	*E*_1_	2.0	GPa
	*b*	20	mm
	$h_{1}$	5	mm
	l	65	mm
	$l_{1}$	10	mm
	*j*	3	
	*S*	11	
PZT-5H	$\rho_{2}$	7500	kg/m^3^
	$s_{11}^{E}$	1.65 × 10^−11^	m^3^/N
	$\varepsilon_{33}^{T}$	3.01 × 10^−8^	F/m
	$d_{31}$	−2.74 × 10^−10^	C/m^2^
	$l_{2}$	45	mm
	$h_{2}$	0.1	mm
	$h_{3}$	0.5	mm
	$\Delta h_{3}$	0.4	mm
Circuit	*L*	0.33	H
	*R*	100	kΩ

**Table 2:** Environment settings of finite element simulation analysis.

Item	Gradient Mode
Spatial dimension	2D
Physical field	Solid Mechanics-Piezoelectric Devices-Circuits
Research type	Frequency domain analysis
*S*	11
*j*	3
*q*	1/5, 1/4, 1/3, 1/2, 1, 2, 3, 4, 5, uniform
Loading method	The acceleration amplitude of the left-hand end face is 10 g
Polarization direction	Vertical upward
Piezoelectric bimorph connection type	Parallel connection
Circuits	*L-R* parallel connection

## Data Availability

The original contributions presented in this study are included in the article. Further inquiries can be directed to the corresponding author.
